# SOCIAL STRATIFICATION AND TRAJECTORIES OF LIVING ARRANGEMENTS AMONG OLDER AMERICANS

**DOI:** 10.1093/geroni/igz038.1038

**Published:** 2019-11-08

**Authors:** Jersey Liang, BoRin Kim, Xiao Xu, James Raymo, Mary Beth Ofstedal, Qing Zheng

**Affiliations:** 1 University of Michigan, Ann Arbor, Michigan, United States; 2 University of New Hampshire, Durham, New Hampshire, United States; 3 Yale University, New Haven, Connecticut, United States; 4 University of Wisconsin-Madison, Madison, Wisconsin, United States; 5 Abt Associates, Durham, North Carolina, United States

## Abstract

Living arrangements are critical to intra-family exchanges that affect older persons’ health and well-being. The conventional conceptualization of living arrangements has emphasized coresidence with children, while overlooking proximate residence from children. Additionally, existing research often relied on cross-sectional data which confound intrapersonal differences with interpersonal variations. This study examined the dynamics of living arrangements in old age by depicting their trajectories as a function of social stratification (i.e., age, gender, race/ethnicity, education, income, and wealth). Data came from the Health and Retirement Study and included a national sample of 7,822 older Americans with at least one living child from 1998 to 2014. Multi-level mixed effects models were employed to analyze the trajectories of living arrangements and their key determinants for the young-old and the old-old separately. Among the young-old (age 65-74, N=4,917), the probability of coresidence increased slightly over time, whereas the probabilities of proximate residence and distant residence decreased slightly and remained stable respectively, and the risk for institutionalization increased moderately. Similar but more accelerated trajectories were observed among the old-old (age 75+, N=2,905). Age, gender, race/ethnicity, education, income, and asset were significantly associated with not only the levels of the probabilities of various living arrangements but also their slopes. For instance, among the old-old, Hispanics had a lower level of nursing home residence as well as a slower rate of increase in the risk of institutionalization than Whites. These findings may inform public policies to strengthen family-based support and long-term care for older people.

